# Expression of CCL21 in Ewing sarcoma shows an inverse correlation with metastases and is a candidate target for immunotherapy

**DOI:** 10.1007/s00262-016-1862-1

**Published:** 2016-07-01

**Authors:** Laurens G. L. Sand, Dagmar Berghuis, Karoly Szuhai, Pancras C. W. Hogendoorn

**Affiliations:** 1 Department of Pathology, Leiden University Medical Center, P.O. Box 9600, 2300 RC Leiden, The Netherlands; 2 Department of Pediatrics, Leiden University Medical Center, Leiden, The Netherlands; 3 Department of Molecular Cell Biology, Leiden University Medical Center, Leiden, The Netherlands

**Keywords:** Bone tumor, Soft tissue tumor, Immunotherapy, Tumor microenvironment, Immune response

## Abstract

Ewing sarcoma is an aggressive neoplasm predominantly occurring in adolescents and has a poor prognosis when metastasized. For patients with metastatic disease in particular, immunotherapy has been proposed as possible beneficial additive therapy. CCL21 activation-based immunotherapy was successful in preclinical studies in other tumor types; therefore, we investigated CCL21 expression in Ewing sarcoma as potential target for immunotherapy. The *CCL21* RNA expression was determined in 21 Ewing sarcoma cell lines and 18 primary therapy-naive Ewing sarcoma samples. In the tumor samples, this was correlated with the number and CD4^+^/CD8^+^ ratio of infiltrating T cells and clinical parameters. Higher RNA expression levels of *CCL21* significantly correlated with a lower CD4^+^/CD8^+^ T cell ratio (*P* = 0.009), good chemotherapeutic response (*P* = 0.01) and improved outcome (*P* < 0.001). In patients with metastases, *CCL21* expression was significantly lower than in patients without (*P* < 0.0005). *CCL21* expression was significantly higher in Ewing sarcoma tissue samples compared to cell lines (*P* < 0.01), implying the involvement of a stromal factor. Protein expression analysis of CCL21 and its receptor CCR7 in 24 therapy-naïve tumors showed that there was no expression in all bar one Ewing sarcoma cells. In conclusion, CCL21 is expressed in clinical Ewing sarcoma samples by nontumor-infiltrating immune cells. The observed positive correlation with survival implies that CCL21 might be a potential prognostic marker for Ewing sarcoma and marks the potential of CCL21 immunotherapy for use in Ewing sarcoma.

**Electronic supplementary material:**

The online version of this article (doi:10.1007/s00262-016-1862-1) contains supplementary material, which is available to authorized users.

## Introduction

Ewing sarcoma (EWS) is the third most common primary bone sarcoma which predominantly occurs in children and adolescents [1]. It is characterized by aggressive/destructive local growth and has a high-grade malignant behavior, with (micro-) metastases at the time of presentation being common. Patients with metastases or recurrent disease have a poor outcome with 15–30 % long-term survival [2, 3].

To date, after the initial introduction of multimodal chemotherapy, no further improvement in survival of these patients has been accomplished, and besides the classical parameters such as tumor site, resectability, response to chemotherapy and size, no prognostic markers are in clinical use for decision making. EWS has a very low number of mutations compared to other tumors, which suggests that corrective apoptosis pathways are still functional, such as TNF-related apoptosis-inducing ligand (TRAIL) pathway [4–6]. The death receptor pathways and other apoptotic pathways are active in EWS and consequently the tumor is sensitive for activation of these mechanisms by natural killer (NK) cells and cytotoxic T cells [7–9]. Immunotherapy in Ewing sarcoma has been shown to have a promising potential role in vitro and is being tested in two clinical trials by administrating donor NK-cells (NCT01287104, NCT02100891) [7, 8, 10].

We previously investigated the immune microenvironment in EWS and demonstrated a relation between the number of infiltrating cytotoxic T cells and patient outcome [11]. Expression levels of pro-inflammatory chemokines [particularly chemokine (C-X-C motif) ligand 9 (CXCL9), chemokine (C-X-C motif) ligand 10 (CXCL10) and chemokine (C-C motif) ligand 5] correlated positively with the number of infiltrating CD8^+^ T cells [11]. Another potent T cell chemoattractant is chemokine (C-C motif) ligand 21 (CCL21), which acts via its receptor chemokine (C-C motif) receptor 7 (CCR7) as a single attractant or in combination with CXCL9 and CXCL10 [12, 13]. In addition, CCL21 may increase dendritic cell-provoked T cell responses, leading to more efficient anti-tumor immune responses [14, 15]. Successful use of CCL21 as immunotherapy has been demonstrated and a trial using dendritic cells expressing CCL21 showed better results than CCL21 used alone in nonsmall lung cancer [16]. Due to the immunogenic role of CCL21 and its immunotherapeutic potential, we studied the CCL21 expression in primary therapy-naïve Ewing sarcoma samples and EWS cell lines by analyzing the RNA expression levels of *CCL21*. The measured RNA expression levels were correlated with the number of infiltrating T cells and the CD4^+^/CD8^+^ T cell ratio in Ewing sarcoma samples. A reversed CD4^+^/CD8^+^ T cell ratio has been reported as predictor of improved outcome in other tumors [17, 18]. In our study, the CD4^+^/CD8^+^ T cell ratio showed inverse correlation with the *CCL21* expression level, and increased *CCL21* expression levels were associated with better survival. This correlation suggests that testing for CCL21 levels in therapy-naïve EWS tumor samples could be used as a prognostic marker and supports a potential role for this cytokine in anti-tumor immunity.

## Materials and methods

### Clinical information on patient samples

Eighteen cryopreserved primary therapy-naïve samples from 18 EWS patients, all containing more than 80 % tumor cells as assessed by light microscopy, and a validation tissue microarray (TMA) of formalin-fixed paraffin-embedded (FFPE) specimens of 16 tumors of 16 patients were obtained from the Department of Pathology, Leiden University Medical Center, and were handled in a coded fashion, according to the Dutch National Ethical Guidelines (‘Code for Proper Secondary Use of Human Tissue’). Ewing sarcoma diagnosis was established according to WHO criteria, including immunohistochemistry (IHC) and *Ewing sarcoma breakpoint region 1 (EWSR1)* translocation detection either by real-time quantitative reverse transcriptase PCR (RT-Q-PCR) or by interphase fluorescence in situ hybridization (FISH). A good chemotherapeutic response was defined by <10 % morphologically viable tumor cells upon histopathologic evaluation of the post-chemotherapy resection specimen [19, 20]. Median patient age at diagnosis of the cohort was 17.5 years (range of 5–35 years) (*Supplementary Table S1*).

### Ewing sarcoma cell lines

Ewing sarcoma cell lines (*n* = 21) were obtained from multiple sources: L-1062 and L-872 were established in-house [21]; CHP100, RM-82, IARC-EW7, TC32 and 6647, CHP100, RM-82, IARC-EW-7, WE-68, IARC-EW-3, STA-ET-2.1, TTC-466, STA-ET-10, CADO-ES1, TC-71, VH-64, COH and STA-ET-1 were obtained from the EuroBoNeT consortium collection (Institute of Pathology, University Medical Center, Düsseldorf, Germany) [22] and SK-ES-1, SK-NM-C, A-673 and R-D-ES from the American Type Culture Collection (ATCC). All cell lines and primary culture L-4027 were cultured in a monolayer under equal conditions and in Iscove’s modified Dulbecco’s medium containing GlutaMAX supplement, supplemented with 1 % streptomycin/penicillin and 10 % heat-inactivated FCS (all from Life Technologies, Bleiswijk, The Netherlands). Authentication of cell lines using Powerplex 1.2 and CellID STR (Promega, Leiden, the Netherlands) and mycoplasma DNA Q-PCR screening were regularly performed on all cell lines.

### RNA isolation

Total RNA was isolated using TRIzol reagent (Life Technologies, Bleiswijk, the Netherlands) according to the manufacturer’s instructions. RNA concentration was measured using Nanodrop, and quality of the RNA was determined using Bioanalyzer2000 RNA Nano chip (Agilent Technology, Amstelveen, The Netherlands). Samples with a RNA integrity number ≥5 were included for RT-Q-PCR analysis.

### RT-Q-PCR analysis and Fluidigm

cDNA generation and RT-Q-PCR using Fluidigm BioMark system was performed according to the H format protocol of the manufacturer (QIAGEN, Venlo, the Netherlands). Samples were prepared for RT-Q-PCR using a 96 × 96 dynamic array chip and performed using BioMark HD system (Fluidigm, San Francisco, CA, USA). All primers for this array chip were obtained from QIAGEN (Venlo, The Netherlands) including nine control genes: *RPL13A, BTF3, YWHAZ, UBE2D2, ATP6V1G1, IPO8, HBS1L*, *AHSP* and *TBP*. Samples were measured in duplicates and analyzed using BioMark software, delivered with the HD system.

### Detection of infiltrating T-lymphocytes

Number of CD4- and CD8-positive T cells were determined according to Berghuis et al. [11]. In brief, FFPE tumor sections were stained for CD3 (Dako, Heverlee, Belgium), CD4 and CD8 (Novocastra, Newcastle upon Tyne, UK) and scanned with Zeiss LSM-510 confocal microscope (Carl Zeiss AG, Göttingen, Germany). In each section 10 areas were selected, digitally photographed and lymphocytes were counted.

### Immunohistochemistry

Tumor sections were stained with anti-CCL21 (clone: HPA051210) (Sigma-Aldrich, Steinheim, Germany) and CCR7 (Abcam, Cambridge, UK) antibodies. Extensive validation data for anti-CCL21 antibody (HPA051210) using IHC on various TMAs and western blots are accessible at the Human Protein Atlas portal [23]. Sections were dewaxed, rehydrated and were subjected to citrate pH6.0 (CCL21) or Tris/HCl-EDTA pH9 (CCR7) antigen retrieval. Sections stained for CCL21 expression were incubated with 5 % nonfat dry milk for 30 min at room temperature and incubated with anti-CCL21 (1:600) in 5 % ELK overnight at 4  °C. Sections stained for CCR7 expression were incubated 1.5 % BSA with anti-CCR7 (1:2000) overnight at 4  °C. Afterward sections were incubated with Immunologic Poly-HRP-GAM/R/R IgG (Leica Biosystems, Eindhoven, The Netherlands) and Dako liquid DAB^+^ Substrate-Chromogen System (Dako, Heverlee, Belgium). Scanning of the slides was performed by Philips Ultra Fast Scanner (Philips Healthcare, Eindhoven, Netherlands). Tonsil tissues, both regular and decalcified FFPE processed, were used as a control. All slides were evaluated by at least two experienced persons of whom one was a reference pathologist (PCWH).

### Statistical analysis

Survival curves were calculated using the Kaplan–Meier method, and *P* values were calculated using the log-rank test using SPSS 20 (IBM Inc. Amsterdam, The Netherlands) and Prism GraphPad 6 (GraphPad Software Inc. La Jolla, CA, USA). Multivariate analysis of the parameters could not be performed due to the limited number of samples. Correlations were calculated with SPSS 20 using Pearson or Spearman correlation. High RNA expression was set as expression above the median. Student *t* test’s *P* value was calculated using Prism GraphPad 6 assuming nonparametric distribution due to limited number of samples and was corrected using Manley-Welch correction.

## Results

**Fig. 1:**
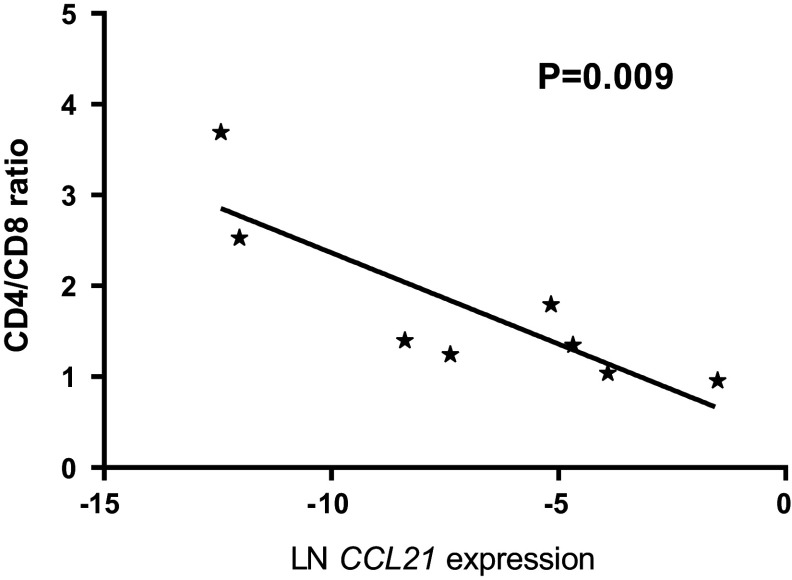
Increased *CCL21* RNA expression correlates with reversed CD4^+^/CD8^+^ ratio of infiltrating CD3^+^ T cells. *CCL21* RNA expression levels of samples with available high-quality RNA and high-quality FFPE material (*n* = 8) were natural log-transformed and correlated with the ratio between the total counted CD3^+^CD4^+^ and CD3^+^CD8^+^-infiltrating T cells. *P* value of the linear regression analysis was demonstrated

**Fig. 2:**
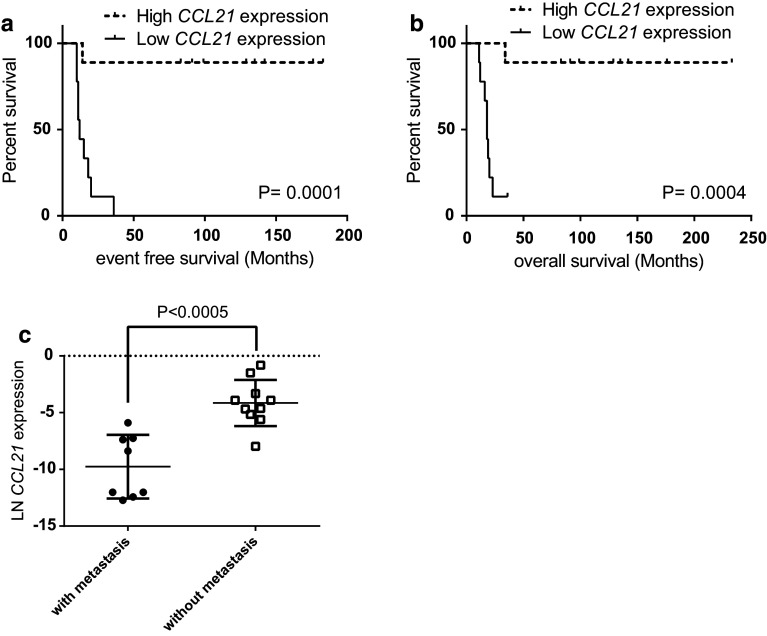
High *CCL21* expression correlated to better EFS and OS. **a**, **b** *CCL21* RNA expression levels of the primary therapy-naïve tumors samples were correlated to EFS and OS using Kaplan–Meier survival analysis. Median was set as threshold to determine high (*dotted line*) and low (*straight line*) *CCL21* expression. **c** Natural log-transformed *CCL21* expression levels were compared between patients who developed a metastasis (+) and patients who did not develop a metastasis (−)

RNA expression of *CCL21* was analyzed in 18 primary therapy-naïve tumor samples, and the expression levels were correlated with the immunohistochemical staining of the CD4^+^- and CD8^+^-infiltrating T cells in eight tissue samples for which sufficient FFPE material was still available (*Supplementary Table S2*). In these samples, the *CCL21* expression was inversely correlated to CD4^+^/CD8^+^ T cell ratio (Fig. 1). However, the absolute numbers of CD8^+^ or CD4^+^ T cells did not correlate with CCL21 expression and varied widely between the samples (data not shown). Since a high-CD8^+^ T cells infiltration was associated in Ewing sarcoma with a better outcome, we correlated *CCL21* RNA expression levels in therapy-naïve tumor samples with development of metastases, survival and chemotherapeutic response. Kaplan–Meier survival analysis demonstrated that an increased *CCL21* expression correlated significantly both with improved-event-free survival (EFS) and with overall survival (OS) (*P* = 0.0001; *P* = 0.0004) (Fig. 2a, b). Moreover, natural logarithm-transformed *CCL21* expression was significantly higher in patient who did not develop a metastasis compared to patients who did (*P* < 0.0005) (Fig. 2c). However, no correlation with metastasis at diagnosis was observed (data not shown). The improved survival may be linked to a better chemotherapeutic response as correlation between good response and increased *CCL21* expression was observed (*P* = 0.02). It should also be noted that good response to chemotherapy was correlated with improved outcome (*P* = 0.008).

**Fig. 3:**
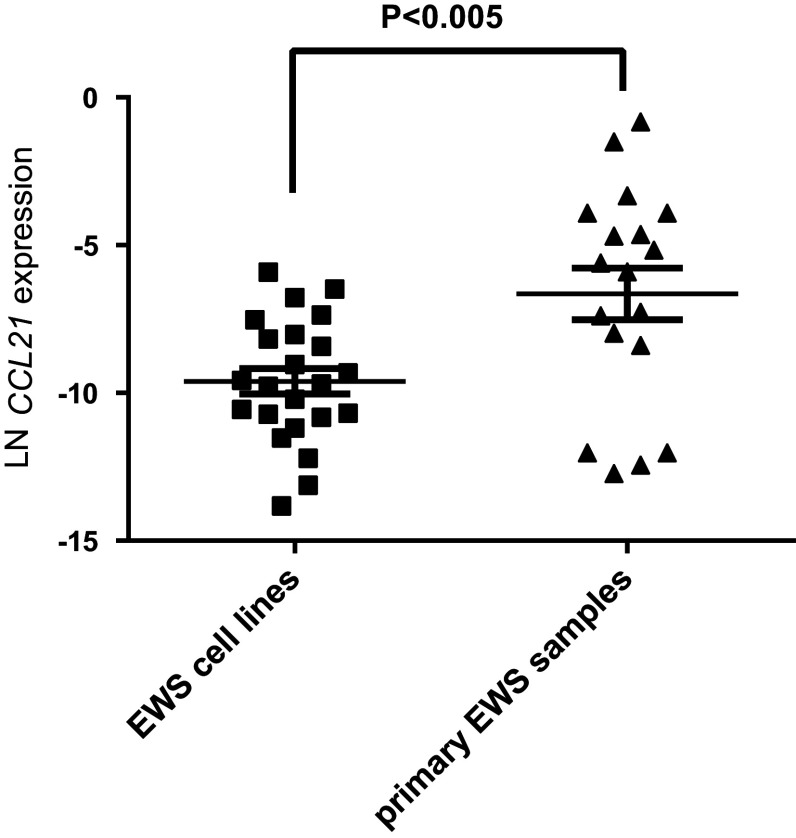
RNA expression levels of *CCL21* were significantly higher in tumor samples compared to cell lines. *CCL21* expression levels of 21 cell lines and 1 primary culture were compared to expression levels of the primary therapy-naïve tumor samples

In addition, we investigated the *CCL21* RNA expression in 21 cell lines and 1 primary culture. The *CCL21* expression levels in the cell lines were significantly lower than the in therapy-naive tumor samples (Fig. 3), with a large variation of expression levels between tumor samples compared to cell lines.

**Fig. 4:**
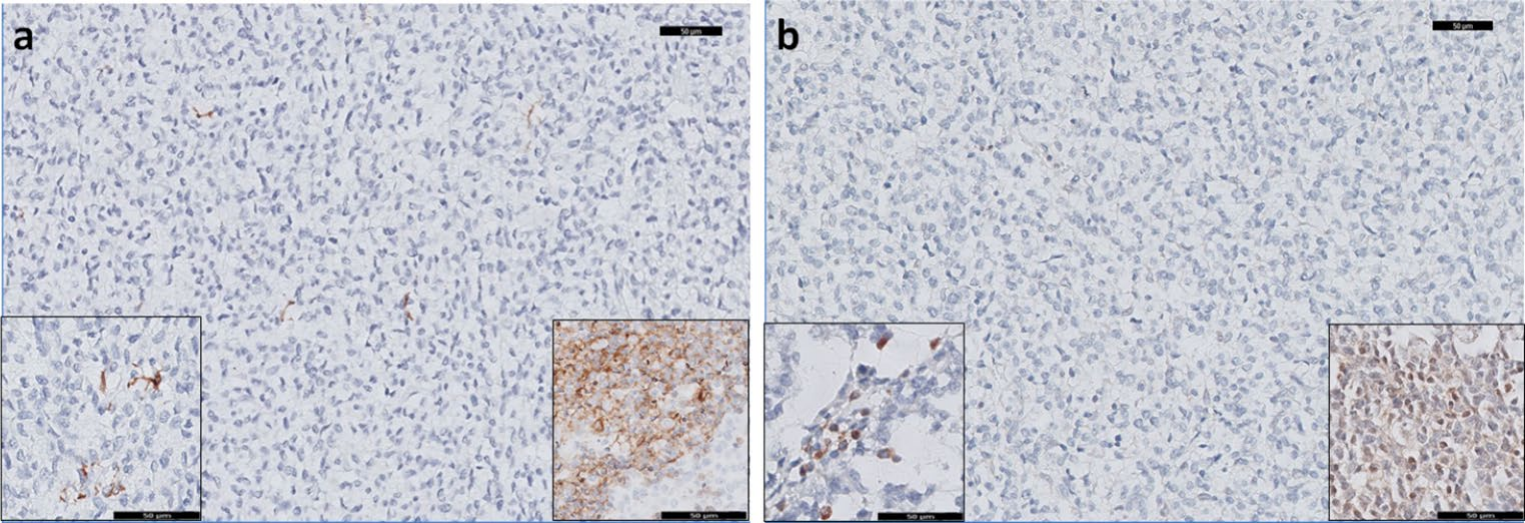
Neither CCR7 nor CCL21 expression was observed in Ewing sarcoma cells by immunohistochemical detection. Eight tumor samples included in the RNA expression analysis and a TMA with 16 samples in duplicate were stained for CCR7 and CCL21 (20× magnification). **a** Tumor cells showed no expression, while infiltrating immune cells showed expression of CCR7 (*left inset*, 40× magnification), positive control is in the *right inset* from tonsil. **b** Tumor cells showed no expression, while infiltrating immune cells showed expression of CCL21 (*left inset*, 40× magnification), positive control is in the *right inset* from tonsil. Magnification: 20×

To show that the difference in expression between tumor samples and cell lines can be accounted for by infiltrating immune cells in the tumor tissues, we studied CCL21 expression at the protein level. The eight cases for which sufficient FFPE material was available were stained for CCL21 using IHC. In addition, the tumor samples were stained for CCR7, the receptor of CCL21. In the tumor samples, EWS cells were negative for CCL21 and CCR7, while infiltrating immune cells did show expression of both CCL21 and CCR7 (Fig. 4). An additional TMA of 16 EWS cases was used for validation of the CCR7 and CCL21 expression pattern. In this TMA, similar to the other cases, EWS cells were CCR7 and CCL21 negative for all but one of the cases.

## Discussion

Previously, we demonstrated that pro-inflammatory chemokines CXCL9 and CXCL10 were associated with an increase in tumor infiltrating CD8^+^ T cells [11]. CCL21 is, like CXCL9 and CXCL10, a CD8^+^ T cell chemoattractant, and its potency is enhanced by the interaction with CXCL9, CXCL10 and interferon gamma (IFNƴ) [13]. These findings prompted us to further investigate the role of CCL21 in EWS. We observed that an increased *CCL21* RNA expression was correlated with a decreased CD4^+^/CD8^+^ ratio. It is likely that these CD3^+^CD8^+^-positive lymphocytes are T cells, but the presence of CD3^+^CD8^+^ NK T cells cannot be excluded [24]. In addition, increased *CCL21* expression correlated with both better EFS and OS and inversely correlated with the development of metastasis. These observations may point toward a role of *CCL21* in the anti-tumor immune response related to the proportion and type of immune cells present in or around the tumor in EWS patients; this has been observed in other tumors including breast cancer and melanoma [25–27]. Even though the presence of infiltrating immune cells in pediatric sarcomas, particularly in EWS, was found to be limited [28], the effect of these cells with regard to therapy response is significant [11].

A second factor which might have had an influence on the observed correlation with patient survival is the chemotherapeutic response of the tumor. In this study (*P* = 0.008) and other studies, a correlation with patient survival was reported [20, 29, 30]. Patients with a good chemotherapeutic response had a higher *CCL21* expression in the tumor sample compared to patients with a poor response. Although it is generally believed that chemotherapy has an immunosuppressive effect by decreasing the number of leukocytes, by now it has become clear that certain chemotherapeutic agents can augment the tumor immunogenicity and stimulate dendritic cell maturation [31–33]. In mice, combining CCL21 immunotherapy with the chemotherapeutic agent paclitaxel had a synergistic effect [34]. CCL21 attracts dendritic cells and is suggested to improve the T cell activation of mature dendritic cells [14, 35]. Increased expression of *CCL21* might be associated with an increase in the number of dendritic cells or an improved immunologic response upon tumor cell death. In patients with CCL21-expressing cells present in or around the tumor, chemotherapy could enhance the anti-tumor immunity and subsequently lead to a better chemotherapeutic response. Our study is based on a small patient cohort, and therefore, a larger study using therapy-naïve samples would be needed to validate the observed correlations.

The significantly higher *CCL21* expression in primary therapy-naïve tumors compared to EWS cell lines suggests the involvement of a stromal factor in *CCL21* expression. CCL21 expression can be enhanced by the interaction with CXCL9, CXCL10 and IFNƴ [13]. However, we reported previously the absence of *CCL21* expression in cell lines even after IFNƴ stimulation indicating that this might be regulated by the EWSR1–friend leukemia virus integration 1 (FLI1) transcription factor [3, 11]. In this study, some cell lines expressed, at low levels, *CCL21*. The difference in CCL21 expression between tumor samples and cell lines might be not only caused by a stromal factor but could also be due to selective in vitro culture conditions. Therefore, the protein expression levels and localization of CCL21 were determined in EWS patient samples using IHC and demonstrated that CCL21 expression was restricted to tumor infiltrating immune cells and that it was not present in EWS cells. In addition, only in one sample CCR7 expression was detected in EWS cells. EWSR1–ETS fusion protein is known to downregulate, directly or indirectly, many chemokines and chemokine receptors, for example by altering regulatory miRNA expression levels and pattern [3]. Of these, the let-7 miRNA family is known to regulate expression of the CCL21–CCR7 [36]. The tumor suppressor let-7a is, for example, known to be directly downregulated by EWSR1–ETS, but this decrease in let-7a does not lead to increased CCR7 expression like in breast cancer cells [37, 38]. Several studies have investigated the role of the let-7 family in EWS and demonstrated a high expression of some members, mainly let-7g, in tumor samples. These studies also revealed various pathways in which these let-7 family members play a role; however, none of them could establish a direct connection between CCL21 or CCR7 and let-7 [37–41].

It is important to note that high CCL21 expression in tumor cells, for example in bladder cancer and breast cancer, is associated with an increased proliferation, number of metastases and a suppressive immune reaction. This might be as a result of paracrine or autocrine activation of a pro-tumorigenic CCL21/CCR7 axis [25, 27, 42]. As in EWS cells, no CCL21 expression was detected and CCR7 expression except in only one sample, and therefore, an active CCL21/CCR7 axis in EWS cells is unlikely. In studies which correlate CCL21 and CCR7 expression, not only the expression level but also the source, tumor cells versus infiltrating immune cells, should also be considered and recorded.

The potency of immunotherapy to treat EWS has been demonstrated by a number of studies [7, 8, 10, 43]. CCL21 is a chemoattractant for dendritic cells, cytotoxic T cells and natural killer cells and can improve the immune response. It has been tested as an immunotherapeutic agent in preclinical and clinical settings as a single agent and combination with chemotherapy [15, 16, 34]. The combination with chemotherapy had a synergistic effect [34]. This could be true for EWS as well, considering the increased expression *CCL21* in patients with a good chemotherapeutic response. However, prior to administration of CCL21 immunotherapy, determination of CCR7 expression in EWS samples may be needed, as high expression of CCL21 and CCR7 expression in tumor cells was found to have negative effect and, one out of the 24 tested EWS samples showed high CCR7 expression in tumor cells. For this case, CCL21 administration might have resulted in an adverse effect, but further studies are needed to draw concrete conclusions. In addition, the potential of CCL21 treatment in not CCL21-primed tumors, meaning no CCL21 expression was present, should be further investigated.

In conclusion, in this study, we showed that patients with increased *CCL21* RNA expression have a better EFS and OS. In addition, protein expression of CCL21 and its receptor CCR7 were not detected in all but one sample of EWS cells, indicating the absence of pro-tumorigenic paracrine and autocrine loops the majority of EWS cases. This tumor entity could therefore serve as a good target for an immunotherapy approach based on the use of CCL21. Furthermore, expression levels of *CCL21* might be used as a potential prognostic marker for survival.

### Electronic supplementary material

Below is the link to the electronic supplementary material.

Supplementary material 1 (PDF 369 kb)

## Abbreviations

ATCC: American type culture collection
CCL21: Chemokine (C-C motif) ligand 21
CCR7: Chemokine (C-C motif) receptor 7
CXCL9: Chemokine (C-X-C motif) ligand 9
CXCL10: Chemokine (C-X-C motif) ligand 10
EFS: Event free survival
ETS: E-twenty-six
EWS: Ewing sarcoma
EWSR1: Ewing sarcoma breakpoint region 1
FFPE: Formalin-fixed paraffin embedded
FISH: Fluorescence in situ hybridization
FLI1: Friend leukemia virus integration 1
IFNy: Interferon gamma
NWO: Netherlands Organization for Scientific Research
RT-Q-PCR: Real-time quantitative reverse transcriptase PCR
TMA: Tissue microarray
TRAIL: TNF-related apoptosis-inducing ligand

## References

[1] De Alava E, Lessnick SL, Sorensen PH, Fletcher CDM, Bridge JA, Hogendoorn PCW, Mertens F (2013). Ewing sarcoma. WHO classification of tumors of soft tissue and bone.

[2] Ladenstein R, Pötschger U, Le Deley MC, Whelan J, Paulussen M, Oberlin O, van den Berg H, Dirksen U (2010). Primary disseminated multifocal Ewing sarcoma: results of the Euro-EWING 99 trial. J Clin Oncol.

[3] Sand LGL, Szuhai K, Hogendoorn PCW (2015). Sequencing overview of Ewing sarcoma: a journey across genomic, epigenomic and transcriptomic landscapes. Int J Mol Sci.

[4] Lawrence MS, Stojanov P, Polak P, Kryukov GV, Cibulskis K, Sivachenko A, Carter SL, Stewart C (2013). Mutational heterogeneity in cancer and the search for new cancer-associated genes. Nature.

[5] Kontny HU, Hammerle K, Klein R, Shayan P, Mackall CL, Niemeyer CM (2001). Sensitivity of Ewing’s sarcoma to TRAIL-induced apoptosis. Cell Death Differ.

[6] Lissat A, Vraetz T, Tsokos M, Klein R, Braun M, Koutelia N, Fisch P, Romero ME (2007). Interferon-γ sensitizes resistant Ewing’s sarcoma cells to tumor necrosis factor apoptosis-inducing ligand-induced apoptosis by up-regulation of caspase-8 without altering chemosensitivity. Am J Pathol.

[7] Verhoeven DHJ, de Hooge ASK, Mooiman ECK, Santos SJ, ten Dam MM, Gelderblom H, Melief CJM, Hogendoorn PCW (2008). NK cells recognize and lyse Ewing sarcoma cells through NKG2D and DNAM-1 receptor dependent pathways. Mol Immunol.

[8] Pahl JW, Ruslan SE, Kwappenberg KC, van Ostaijen-ten Dam M, van Tol MD, Lankester A, Schilham M (2013). Antibody-dependent cell lysis by NK cells is preserved after sarcoma-induced inhibition of NK cell cytotoxicity. Cancer Immunol Immunother.

[9] de Hooge ASK, Berghuis D, Santos SJ, Mooiman E, Romeo S, Kummer JA, Egeler RM, van Tol MJD (2007). Expression of cellular FLICE inhibitory protein, caspase-8, and protease inhibitor-9 in Ewing sarcoma and implications for susceptibility to cytotoxic pathways. Clin Cancer Res.

[10] Evans CH, Liu F, Porter RM, O’Sullivan RP, Merghoub T, Lunsford EP, Robichaud K, Van Valen F (2012). EWS-FLI-1-targeted cytotoxic T cell killing of multiple tumor types belonging to the Ewing sarcoma family of tumors. Clin Cancer Res.

[11] Berghuis D, Santos SJ, Baelde HJ, Taminiau AHM, Egeler MR, Schilham MW, Hogendoorn PCW, Lankester AC (2011). Pro-inflammatory chemokine–chemokine receptor interactions within the Ewing sarcoma microenvironment determine CD8 + T-lymphocyte infiltration and affect tumour progression. J Pathol.

[12] Lo JC, Chin RK, Lee Y, Kang H-S, Wang Y, Weinstock JV, Banks T, Ware CF (2003). Differential regulation of CCL21 in lymphoid/nonlymphoid tissues for effectively attracting T cells to peripheral tissues. J Clin Invest.

[13] Sharma S, Yang S-C, Hillinger S, Zhu LX, Huang M, Batra RK, Lin JF, Burdick MD (2003). SLC/CCL21-mediated anti-tumor responses require IFNγ, MIG/CXCL9 and IP-10/CXCL10. Mol Cancer.

[14] Hong CY, Lee HJ, Kim HJ, Lee JJ (2014). The lymphoid chemokine CCL21 enhances the cytotoxic T lymphocyte-inducing functions of dendritic cells. Scand J Immunol.

[15] Lin Y, Sharma S, John MS (2014). CCL21 Cancer Immunotherapy. Cancers (Basel).

[16] Lee JM, Garon EB, Lee M, Baratelli F, Wang G, Abtin F, Suh R, Wallace WD (2014). Phase I trial of trans-thoracic injection of CCL21 gene modified dendritic cells in human non-small cell lung carcinoma. J Surg Res.

[17] Shah W, Yan X, Jing L, Zhou Y, Chen H, Wang Y (2011). A reversed CD4/CD8 ratio of tumor-infiltrating lymphocytes and a high percentage of CD4(+)FOXP3(+) regulatory T cells are significantly associated with clinical outcome in squamous cell carcinoma of the cervix. Cell Mol Immunol.

[18] García-Martínez E, Gil GL, Benito AC, González-Billalabeitia E, Conesa MAV, García TG, García-Garre E, Vicente V (2014). Tumor-infiltrating immune cell profiles and their change after neoadjuvant chemotherapy predict response and prognosis of breast cancer. Breast Cancer Res.

[19] van der Woude HJ, Bloem JL, Holscher HC, Nooy MA, Taminiau AHM, Hermans J, Falke THM, Hogendoorn PCW (1994). Monitoring the effect of chemotherapy in Ewing’s sarcoma of bone with MR imaging. Skelet Radiol.

[20] Picci P, Rougraff BT, Bacci G, Neff JR, Sangiorgi L, Cazzola A, Baldini N, Ferrari S (1993). Prognostic significance of histopathologic response to chemotherapy in nonmetastatic Ewing’s sarcoma of the extremities. J Clin Oncol.

[21] Szuhai K, Ijszenga M, Tanke HJ, Rosenberg C, Hogendoorn PCW (2006). Molecular cytogenetic characterization of four previously established and two newly established Ewing sarcoma cell lines. Cancer Genet Cytogenet.

[22] Ottaviano L, Schaefer K-L, Gajewski M, Huckenbeck W, Baldus S, Rogel U, Mackintosh C, de Alava E (2010). Molecular characterization of commonly used cell lines for bone tumor research: a trans-European EuroBoNet effort. Genes Chromosom Cancer.

[23] Pontén F, Jirström K, Uhlen M (2008). The Human Protein Atlas—a tool for pathology. J Pathol.

[24] Lanier LL, Chang C, Spits H, Phillips JH (1992). Expression of cytoplasmic CD3 epsilon proteins in activated human adult natural killer (NK) cells and CD3 gamma, delta, epsilon complexes in fetal NK cells. Implications for the relationship of NK and T lymphocytes. J Immunol.

[25] Tutunea-Fatan E, Majumder M, Xin X, Lala PK (2015). The role of CCL21/CCR7 chemokine axis in breast cancer-induced lymphangiogenesis. Mol Cancer.

[26] Shields JD, Emmett MS, Dunn DBA, Joory KD, Sage LM, Rigby H, Mortimer PS, Orlando A (2007). Chemokine-mediated migration of melanoma cells towards lymphatics—a mechanism contributing to metastasis. Oncogene.

[27] Shields JD, Kourtis IC, Tomei AA, Roberts JM, Swartz MA (2010). Induction of lymphoid like stroma and immune escape by tumors that express the chemokine CCL21. Science.

[28] Vakkila J, Jaffe R, Michelow M, Lotze MT (2006). Pediatric cancers are infiltrated predominantly by macrophages and contain a paucity of dendritic cells: a major nosologic difference with adult tumors. Clin Cancer Res.

[29] Paulussen M, Ahrens S, Dunst J, Winkelmann W, Exner GU, Kotz R, Amann G, Dockhorn-Dworniczak B (2001). Localized Ewing tumor of bone: final results of the cooperative Ewing’s Sarcoma Study CESS 86. J Clin Oncol.

[30] Oberlin O, Deley MCL, Bui BN, Gentet JC, Philip T, Terrier P, Carrie C, Mechinaud F (2001). Prognostic factors in localized Ewing’s tumours and peripheral neuroectodermal tumours: the third study of the French Society of Paediatric Oncology (EW88 study). Br J Cancer.

[31] Zitvogel L, Apetoh L, Ghiringhelli F, Kroemer G (2008). Immunological aspects of cancer chemotherapy. Nat Rev Immunol.

[32] Ma Y, Adjemian S, Mattarollo Stephen R, Yamazaki T, Aymeric L, Yang H, Catani JP, Hannani D (2013). Anticancer chemotherapy-induced intratumoral recruitment and differentiation of antigen-presenting cells. Immunity.

[33] Emens LA, Middleton G (2015). the interplay of immunotherapy and chemotherapy: harnessing potential synergies. Cancer Immunol Res.

[34] Chen P, Luo S, Wen Y-J, Li Y-H, Li J, Wang Y-S, Du L-C, Zhang P (2014). Low-dose paclitaxel improves the therapeutic efficacy of recombinant adenovirus encoding CCL21 chemokine against murine cancer. Cancer Sci.

[35] Murphy PM (2010). Double duty for CCL21 in dendritic cell trafficking. Immunity.

[36] Kim S-J, Shin J-Y, Lee K-D, Bae Y-K, Sung K, Nam S, Chun K-H (2012). MicroRNA let-7a suppresses breast cancer cell migration and invasion through downregulation of C-C chemokine receptor type 7. Breast Cancer Res.

[37] De Vito C, Riggi N, Suvà M-L, Janiszewska M, Horlbeck J, Baumer K, Provero P, Stamenkovic I (2011). Let-7a is a direct EWS-FLI-1 target implicated in Ewing’s sarcoma development. PLoS ONE.

[38] Zhang Z, Huang L, Yu Z, Chen X, Yang D, Zhan P, Dai M, Huang S (2014). Let-7a functions as a tumor suppressor in Ewing’s sarcoma cell lines partly by targeting cyclin-dependent kinase 6. DNA Cell Biol.

[39] Karnuth B, Dedy N, Spieker T, Lawlor ER, Gattenlöhner S, Ranft A, Dirksen U, Jürgens H (2014). Differentially expressed miRNAs in Ewing sarcoma compared to mesenchymal stem cells: low miR-31 expression with effects on proliferation and invasion. PLoS ONE.

[40] Sohn EJ, Park J, Kang S-i, Wu Y-P (2012). Accumulation of pre-let-7 g and downregulation of mature let-7 g with the depletion of EWS. Biochem Biophys Res Commun.

[41] Hameiri-Grossman M, Porat-Klein A, Yaniv I, Ash S, Cohen IJ, Kodman Y, Haklai R, Elad-Sfadia G (2015). The association between let-7, RAS and HIF-1α in Ewing sarcoma tumor growth. Oncotarget.

[42] Mo M, Zhou M, Wang L, Qi L, Zhou K, Liu L-F, Chen Z, Zu X-B (2015). CCL21/CCR7 enhances the proliferation, migration, and invasion of human bladder cancer T24 cells. PLoS ONE.

[43] Berghuis D, Schilham MW, Vos HI, Santos SJ, Kloess S, Buddingh' EP, Egeler RM, Hogendoorn PCW (2012). Histone deacetylase inhibitors enhance expression of NKG2D ligands in Ewing sarcoma and sensitize for natural killer cell-mediated cytolysis. Clin Sarcoma Res.

