# Supplementary material for: Expression of CCL21 in Ewing sarcoma shows an inverse correlation with metastases and is a candidate target for immunotherapy
Source: Cancer Immunol Immunother. 2016 Jul 1;65(8):995–1002. doi: 10.1007/s00262-016-1862-1 (PMC4956712; doi:10.1007/s00262-016-1862-1)
Supplement: Supplementary file 1 — Supplementary material 1 (PDF 369 kb) [file 262_2016_1862_MOESM1_ESM.pdf]

Supplementary table S1. Clinical details of study patients of the primary therapy-naïve tumor samples.

Patient number	Age (yrs)	Sex	Primary tumor site	Metastasis at diagnosis[a]	Starting treatment protocol	Response to chemotherapy[b]	Translocation	Metastasis later[c]	Relapse[d]	EFS Time (month)	EFS[e]	OS Time (month)	OS[f]
L318	35	male	proximal radius	0	CESS86	1	EWS-FLI1	0	0	183	0	233	0
L463	24	male	thorax wall	0	CESS86	ND	EWS-FLI1	1	1	12	1	20	1
L469	19	female	distal fibula	0	EICESS	0	EWS-FLI1	1	1	20	1	23	1
L513	11	male	pelvis	1	EICESS	ND	EWS-FLI1	0	ND	18	1	18	1
L683	17	male	tibia	0	EICESS	0	EWS-FLI1	1	0	10	1	16	1
L848	15	female	humerus	1	EuroEwing99	1	EWS-FLI1	0	0	142	0	142	0
L1016	5	male	tibia + fibula	1	EuroEwing99	1	Clinical Ewing sarcoma*	0	0	135	0	135	0
L1034	18	male	pelvis	1	EuroEwing99	0	EWS-FLI1	1	0	11	1	18	1
L1098	10	male	femur	0	EuroEwing99	1	EWS-FLI1	0	0	129	0	129	0
L1220	19	male	os pubis	1	EuroEwing99	ND	EWS-FLI1	1	0	10	1	11	1
L1232	14	male	humerus	0	EuroEwing99	ND	EWS-FLI1	0	ND	14	1	34	1
L1379	13	male	fibula	1	EuroEwing99	0	EWS-FLI1	0	0	99	0	99	0
L1489	25	male	pelvis	0	EuroEwing99	1	EWS break by FISH	0	0	91	0	91	0
L1570	12	male	humerus	0	EuroEwing99	1	EWS-FLI1	0	0	83	0	83	0
L1722	18	male	humerus	0	EuroEwing99	1	EWS-FLI1	1	1	36	1	36	0
L2154	11	female	femur	1	EuroEwing99	1	EWS-FLI1	0	0	176	0	176	0
L2161	19	male	pelvis	0	EuroEwing99	0	EWS-FLI1	1	0	11	1	12	1
L2162	19	male	pelvis	1	EuroEwing99	ND	EWS-FLI1	1	0	15	1	19	1

ND: Not determined

EFS: Event free survival

OS: Overall survival

[a,c,d,e] **1:** Event reported or **0**: No event reported

[b] **1**: < 10% tumor vitality or **0** > 10% tumor vitality

[f] **1:** Dead or **0:** Alive.

*: a case with RT-Q-PCR negativity for *EWSR1-FLI1*, *EWSR1-ERG* in diagnostic settings, negativity for *BCOR-CCNB3* RT-Q-PCR and the lack of *EWSR1*, *FUS* and *CIC* split apart signals from FFPE sections. Clinical as well as morphological and immunohistochemically consistent with Ewing Sarcoma.

Supplementary table S2. Counts of T-cells and *CCL21* RNA expression levels

Patient	Normalized *CCL21* RNA expression	Tumor infiltrating T-cells (cells/mm2)		
		$CD3^+CD4^+$	$CD3^+CD8^+$	$CD4^+/CD8^+$ ratio
L2162	0.000004	118	32	3.68
L1722	0.000006	96	38	2.53
L1220	0.00023	49	35	1.40
L1034	0.00063	61	49	1.24
L1570	0.0058	138	77	1.79
L2154	0.0093	155	115	1.35
L1098	0.020	26	25	1.04
L1489	0.22	91	95	0.96
